# Associations between RetNet gene polymorphisms and the efficacy of orthokeratology for myopia control: a retrospective clinical study

**DOI:** 10.1186/s40662-025-00426-4

**Published:** 2025-03-17

**Authors:** Ruijing Xia, Xiangyi Yu, Hao Wu, Lulu Peng, Zhenlin Du, Xiaoguang Yu, Shilai Xing, Fan Lu, Xinjie Mao

**Affiliations:** 1 National Clinical Research Center for Ocular Diseases, Eye Hospital, Wenzhou Medical University, Wenzhou, 325027 China; 2 Institute of PSI Genomics Co., Ltd., Shanghai, China

**Keywords:** Orthokeratology, Myopia control, WGS, Burden test, Single-variant association analysis

## Abstract

**Background:**

This study investigated how clinical and genetic factors impact the effectiveness of orthokeratology lenses in myopia.

**Methods:**

A retrospective clinical study was conducted with a sample of 545 children aged 8–12 years who had myopia and have initially worn orthokeratology lenses for one year. Whole-genome sequencing (WGS) was also performed on 60 participants in two groups, one with rapid axial length (AL) progression of larger than 0.33 mm and the other with slow AL progression of less than 0.09 mm. The RetNet database was used to screen candidate genes that may contribute to the effectiveness of orthokeratology lenses in controlling myopia.

**Results:**

Children with greater baseline AL, greater spherical equivalent (SE) and greater age had better myopia control with orthokeratology lenses. A significant excess of nonsynonymous variants was observed among those with slow myopia progression, and these were prominently enriched in retinal disease-related genes. Subsequently, *RIMS2* [odds ratio (OR) = 0.01, *P* = 0.0097] and *LCA5* (OR = 9.27, *P* = 0.0089) were found to harbor an excess number of nonsynonymous variants in patients with slow progression of high myopia. Two intronic common variants rs36006402 in *SLC7A14* and rs2285814 in *CLUAP*1 were strongly associated with AL growth. The identification of these novel genes associated with the effectiveness of orthokeratology lens therapy in myopic children provides insight into the genetic mechanism of orthokeratology treatment.

**Conclusion:**

The effectiveness of orthokeratology lens treatment relates to interindividual variability in the control of AL growth in myopic eyes. The efficacy increased when patients carried more nonsynonymous variants in retinal disease-related gene sets. These data serve as reference for genetic counselling and the management of patients who choose orthokeratology lenses to control myopia.

**Supplementary Information:**

The online version contains supplementary material available at 10.1186/s40662-025-00426-4.

## Background

Refractive error, which is the most common visual impairment, is a loss of uncorrected vision as a result of a change in the shape of the eye that prevents light from being accurately refracted and focused on the retina. Due to its increasing prevalence, myopia, a type of refractive error, has become a global public health problem. Globally, 10%–30% of adults suffer from myopia, and in the United States and Europe, the prevalence of myopia among young adults is higher at 40%–50%, with even higher prevalence rates of 80%–90% in some countries in East Asia and Southeast Asia [1–8]. Myopia is also strongly associated with a number of ocular diseases, such as cataracts, glaucoma and myopic macular degeneration [9].

Myopia can usually be corrected by spectacles, contact lenses or refractive surgery to provide good vision. There are many ways to control the progression of myopia [10]. Atropine eye drops, orthokeratology, peripheral defocus-modifying contact lenses or spectacles, and contrast-modifying spectacles are effective at controlling axial length (AL) elongation [11]. Orthokeratology lenses focus light in front of the peripheral retina primarily by changing the curvature of the cornea, thereby refocusing the image centrally on the fovea [12]. This causes the image contour to focus centrally, while creating myopic defocus in the periphery, which is thought to slow the progression of myopia.

However, there are strong individual differences in the control effects of orthokeratology lenses. Some patients have fairly good control effects, while others have very limited control effects or even accelerated regression [13–16]. In several studies, baseline corneal stiffness, lower baseline myopia, younger initial age and higher parental myopia have been identified as factors influencing the effectiveness of orthokeratology lens control [17, 18]. Although many risk factors for the efficacy of orthokeratology lenses for myopia control have been revealed, the genetic factors influencing the effectiveness of orthokeratology lens treatment are still unknown.

This study explored the genetic characteristics of 289 retinal disease-related genes involved in retinal signaling, synaptic function and cell maintenance from the Retinal Information Network database (RetNet, https://retnet.org/, as of September 2024) and the clinical features of a cohort of orthokeratology lens users. Given the importance of the retina in regulating eye growth and its potential role in myopia control, it was hypothesized that variations in these genes could influence how the retina responds to orthokeratology treatment. Hyperopic shift occurs in the central retina and myopic defocus in the peripheral retina after overnight orthokeratology [14, 19, 20], suggesting an inextricable role of retinal photoreceptor mechanisms in orthokeratology lens wear. This study also investigated whether these genes and specific single nucleotide polymorphisms (SNPs) are associated with the effectiveness of orthokeratology lens treatment. These results provide insight into the relationships between genes related to retinal function and the effectiveness of orthokeratology lens treatment, thereby enhancing our understanding of the genetic background of myopia treatment and providing new perspectives for the development of personalized vision correction.

## Methods

### Subjects

This study adopted a retrospective clinic study design. The study protocol received full approval from the institutional ethics committee of Eye Hospital of Wenzhou Medical University with approval number 2023-059-K-48-05. Written informed consent was obtained from each participant. The orthokeratology lenses used in this study included four-zone reverse geometry lenses (Euclid Systems Corp., Herndon, Virginia, USA; LUCID Corp., Fenghua County, Korea) with a nominal Oxygen permeability (Dk) of 95 × 10^−11^ (cm^2^/s) (mL O_2_/mL·mmHg) and 100 × 10^−11^ (cm^2^/s) (mL O_2_/mL·mmHg) [21]. Ocular examinations were performed at baseline and one year after orthokeratology lens wear. Each subject underwent a comprehensive baseline eye examination, including a slit-lamp examination and testing for noncycloplegic subjective manifest refraction and spherical equivalent (SE), uncorrected visual acuity, best-corrected visual acuity, AL (IOL-Master 500, Carl Zeiss Meditec AG, Jena, Germany), corneal topography (E-300, Medmont International Pty. Ltd., Victoria, Australia) and intraocular pressure (Canon TX-20, Canon Inc., Tokyo, Japan). At each follow-up, unaided visual acuity and corneal topography were assessed. All children were treated by doctors who had worked in the field of orthokeratology lenses at the hospital for more than 10 years. The doctor ordered the best lens for the subject based on that subject’s corneal topography and then evaluated the fit of the corneal fluorescein pattern.

**Fig. 1:**
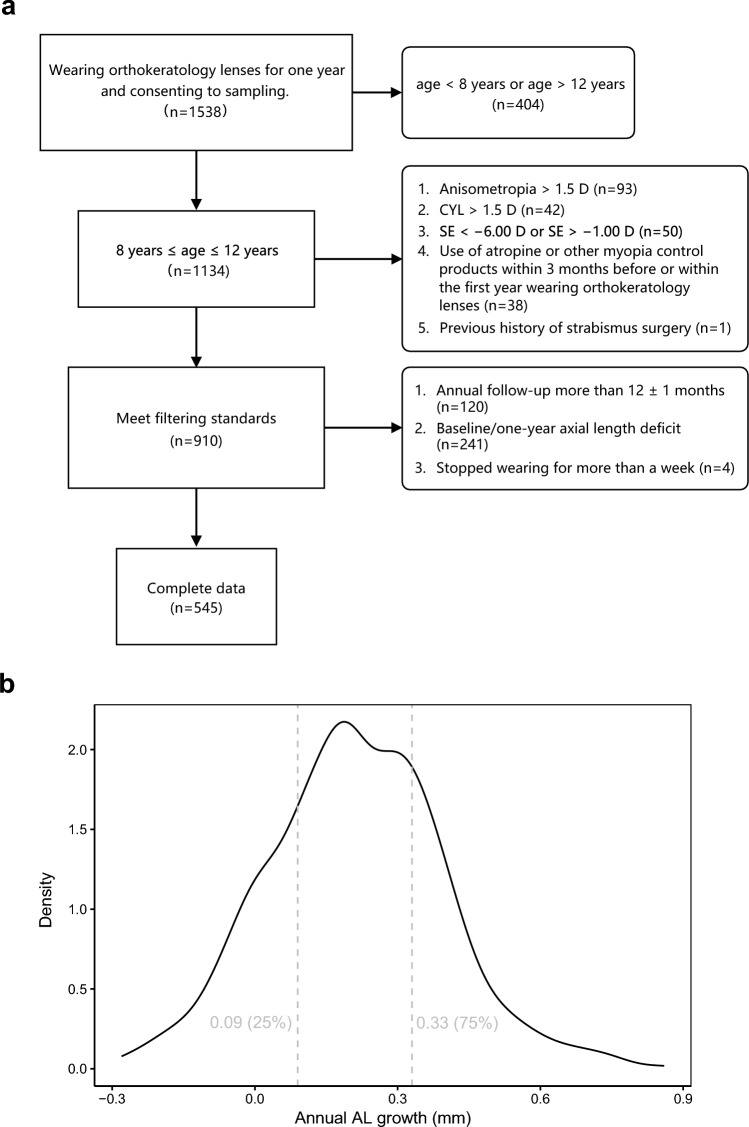
Study flowchart and sample screening process. **a** Screening process for 1538 study participants. **b** Selection of groups with distinct control effects based on quartiles. CYL, cylinder; SE, spherical equivalent

A total of 1,538 myopic patients who were initially wearing orthokeratology lenses were enrolled in the study. These 1,538 patients were critically reviewed and screened, leaving 545 patients with complete data that met the inclusion criteria. Each participant had clinical phenotypic data for both eyes. The amount of AL growth in both eyes of each participant was considered an indication of the effectiveness of the orthokeratology lens in controlling myopia. The amount of AL growth per year was measured in each participant and the upper and lower quartiles of AL growth were taken as the case group (lower myopia progression, annual AL growth ≤ 0.09 mm) and control group (higher myopia progression, annual AL growth ≥ 0.33 mm, Fig. 1b), respectively. In total, 143 cases had an annual AL growth of less than or equal to 0.09 mm and 140 controls had an annual AL growth of greater than or equal to 0.33 mm. After the sample recall process, 30 cases and 30 controls agreed to participate and underwent genetic testing with their written informed consent given. These individuals were included as the final analysis cohort.

### Sequencing and variant calling

The genomic DNA of all subjects was isolated from oral swabs via standard procedures. The method of DNA purification for sequencing is provided in Additional File 1. Whole-genome sequencing (WGS) was performed using DNBSEQ-T7 (BGI, Shenzhen, China). Variant detection and joint genotype calling analyses were conducted based on the Sentieon DNAscope pipeline (Sentieon Inc., version 202308) [22]. The sequence reads of each sample in FASTQ format were aligned against the human reference genome (Genome Reference Consortium Human Build 38 Organism, GRCh38) using the Burrows-Wheeler Aligner (BWA)-MEM [23]. The alignment file was sorted using the Sentieon sort algorithm, and the Sentieon Dedup algorithm was used to mark duplicate reads. Then, SNPs and indels were called in genomic variant call format (GVCF) using Haplotyper. The Sentieon GVCFtyper jointly called subjects as a cohort.

### Quality control

Standard variant-level quality controls were applied. Variants were excluded from further analysis if they had an average genotype depth (DP) < 20 and a genotype quality (GQ) < 40. Population outliers were detected, and stratification was performed using a method based on principal component analysis. The Plink 2.0 [24] (Additional File 2) results indicated that the affected individuals and control subjects were genetically matched for all sequenced samples. Principal components (PCs) 1–10 were assessed for their associations with the disease phenotype status using a generalized linear model (GLM) and were then included in the following analyses as covariates. A population check was conducted on East Asian populations including individuals from CHB (China Beijing), CHS (China South), CDX (Chinese Dai in Xishuanagbanna), JPT (Japan) and KHV (Korean) in the 1000 Genome Project (1KG) [25].

### Variant annotation

The annotation of variants was performed with Ensembl’s Variant Effect Predictor (VEP v.0.1.16) [26] for the human genome assembly GRCh38. Population allele frequency (AF) data from the following databases were used: 1000 Genomes, ESP and Genome Aggregation Database (gnomAD) [27]. Multiple in silico prediction algorithms, including PolyPhen-2 [28], SIFT [29], Combined Annotation Dependent Depletion (CADD) [30], LOFTEE and SpliceAI [31] plugins, were employed to generate additional bioinformatic predictions of variant deleteriousness. Protein-coding variants were annotated into the following three classes: (1) synonymous, (2) nonsynonymous, (3) noncoding.

### Gene-set burden analysis

To estimate the extent to which variants with different allele frequencies and different functions were over-represented in individuals with different control effects, burden tests were conducted across the entire genome and 289 RetNet genes [32]. Common and rare variants were differentiated according to AF from ChinaMap [33], with variants with a minor allele frequency (MAF) less than 0.05 classified as rare variants and vice versa for common variants. For RetNet genes, a logistic test was performed by regressing the case–control status on certain classes of variants aggregated across the target gene set in an individual, with adjustment for sex, age, baseline AL, the top 10 PCs, and the genome-wide variant count.

### Gene-based collapsing analysis

For the gene-based test, testing was restricted to common variants annotated as nonsynonymous. To assess whether a specific gene exhibited an over-representation or under-representation of common nonsynonymous cases, five gene-level association tests were performed, including Fisher’s exact test, logistic, SNP-Set (Sequence) Kernel Association Test (SKAT) [34], SKAT-O [35] and Magma [36], with the previously defined covariates (sex, age, PC1–PC10).

### Cell type enrichment

The single-cell RNA-seq expression matrix was acquired to identify cell-specific biomarkers for investigating the molecular mechanisms underlying complex traits and uncovering previously unrecognized cellular populations that may play important roles in response to orthokeratology treatment [37]. A single-cell RNA-seq (scRNA-seq) expression matrix and metadata on developing human embryonic eyes were acquired from the Broad Institute Single Cell Portal (https://singlecell.broadinstitute.org/, SCP1311) and scRNA-seq analysis was performed in R4.3.2.

### Single-variant association analysis

The associations between common variants (MAF > 0.05) were estimated using Saige [38], fastGWAS [39], PLINK, MLMA-LOCO [40] and EMMAX [41] tests and were corrected for the first 10 PCs.

### Statistical analysis

Multivariable logistic regression models were constructed to evaluate the associations between each factor and change in the AL. Statistical analyses were performed using R4.3.2. The differences in phenotypes and sequencing quality between the groups were compared by Student’s *t*-tests. These phenotypes were also evaluated for phenotype-genotype correlations with the Wilcoxon rank sum test. Statistical significance was defined as a *P* value less than 0.05. *P < 0.05, **P < 0.01, ***P < 0.001.

## Results

### Subject demographics

**Fig. 2:**
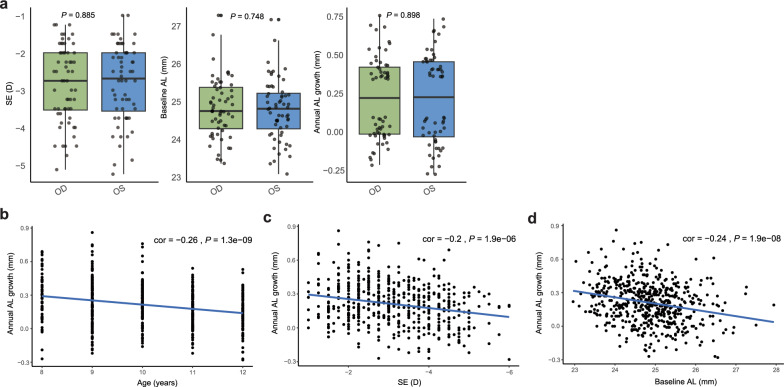
Correlations between baseline data and annual axial length (AL) growth. **a** Baseline differences in spherical equivalent (SE), baseline AL and annual AL growth. Correlation between annual AL growth and (**b**) age, (**c**) SE, (**d**) baseline AL. OD, oculus dexter (right eye); OS, oculus sinister (left eye)

After rigorous review, 545 of the 1538 patients were considered to have met the inclusion criteria and had complete data (Additional File 2). At baseline, their age ranged from 8 to 12 years (10.12 ± 1.27 years), their SE refractive error ranged from − 1.00 to − 6.00 diopters (D) (− 3.11 ± 1.08 D), and their AL ranged from 22.96 to 27.94 mm (24.85 ± 0.80 mm). There was no difference in the amount of annual AL growth between the types of orthokeratology lenses used in this study (Euclid, USA and Lucid, Korea. *P* = 0.66, Wilcoxon rank sum test). There were differences in the clinical data between the left and right eyes, so in the subsequent analysis, the right eye was chosen for analysis (Fig. 2a).

### Effects of clinical factors on the effectiveness of orthokeratology lenses

The correlations between the baseline phenotype (age, SE, AL) and annual AL growth were investigated in the 545 complete samples. Baseline AL was correlated with age (cor = 0.191, *P* = 7.1e−08). Further, all three phenotypes were correlated with the AL growth in both eyes (Fig. 2b, c, d). The older samples had smaller AL growth (cor =  − 0.26, *P* = 1.3e−09). The same negative correlation was found for baseline AL (cor =  − 0.2, *P* = 1.9e−06) and SE (cor =  − 0.24, *P* = 1.9e−08).

**Fig. 3:**
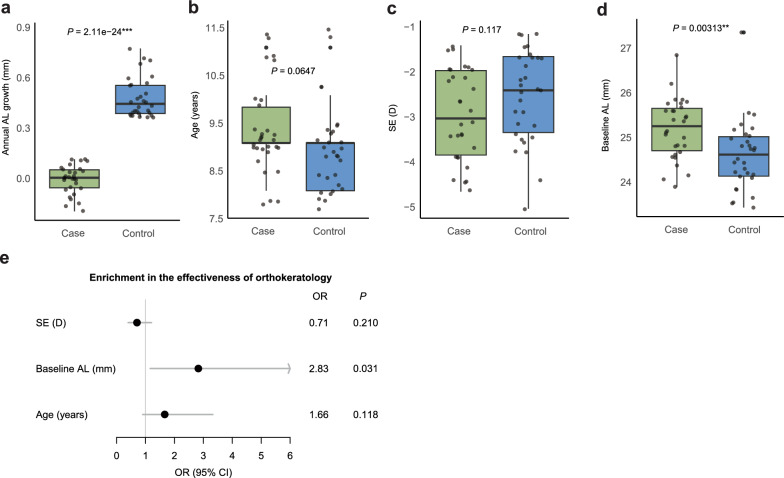
Differences in baseline data and clinical factors associated with control effectiveness. Differences between cases and controls in (**a**) axial length (AL) growth, (**b)** age, (**c**) baseline SE and (**d**) baseline AL. **e** Logistic regression of the effects on orthokeratology lens effectiveness adjusted for age and sex. OR, odds ratio; CI, confidence interval

Further, the baseline phenotypes of 30 samples with well-controlled AL growth and 30 samples with poorly-controlled AL growth were investigated. Significant differences in the amount of AL growth were found between the two groups (*P* = 2.11e−24, Fig. 3a). There were no significant differences in the age distribution or SE between the two groups (Fig. 3b, c). In contrast, a significant difference in the baseline AL was found between the two groups (*P* = 0.00313, Fig. 3d).

The effects of different baseline phenotypes on the orthokeratology lens treatment effect for myopia progression were subsequently tested using a multivariable logistic regression model (Fig. 3e). A significant increase in the baseline AL was observed among cases with a positive orthokeratology lens effect compared to controls [odds ratio (OR) = 2.83, *P* = 0.0308].

### WGS of 60 samples

After stringent quality control, WGS data from 30 cases and 30 controls were analyzed. A total of 7,644,581 biallelic variants were used for further analysis, including 3,233,468 common variants and 3,254,265 rare variants according to ChinaMap. Sequencing quality was not significantly different between the cases and controls (Additional File 2). These remaining samples were all ancestry-matched, closely resembling CHB and CHS ancestry in the 1000G genome.

### Excesses of gene set-based nonsynonymous variants

To aggregate multiple alleles of presumed similar impact in retinal disease-associated genes, a complementary strategy focusing on variants in different functional regions was adopted. The ability to detect variant associations was improved by exploiting the more robust functional annotation of coding variation. The associations among the burden of all variants, common variants, and rare variants were first evaluated by Firth logistic models. Then, the burden test was dissected into the RetNet gene set. Specifically, the model used Firth-based logistic regression and incorporated patient sex, PCs 1–10, the total genome count and patient baseline AL. There were no significant differences in any of the variants between cases and controls when the gene set was not restricted (Additional File 4).

**Fig. 4:**
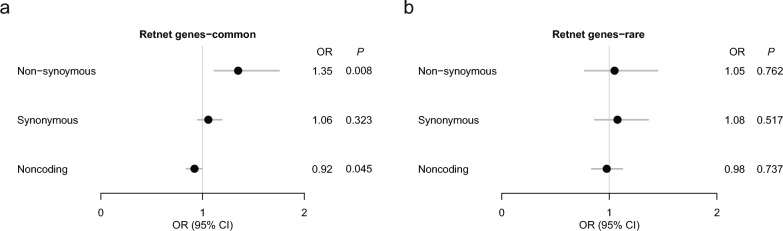
Gene-set polygenic burden test of different types of variants. Gene-set burden analysis of nonsynonymous, synonymous and noncoding variants with (**a**) minor allele frequency (MAF) > 0.05, (**b**) MAF < 0.05. OR, odds ratio; CI, confidence interval

Gene sets covering different biological processes and pre-experimental validations could refine our understanding of the mechanisms underlying the associations between these variants and the control effects of orthokeratology lenses and may help to derive potential biological hypotheses for subsequent detailed analyses. The RetNet gene collection was chosen to explore the associations between retina-associated biological pathway genes and the efficacy of orthokeratology lenses. After restricting the gene set, significant enrichment of nonsynonymous variants was observed in cases (OR = 1.34, *P* = 0.00106, Fig. 4a).

### Gene-based common variant association analysis

**Fig. 5:**
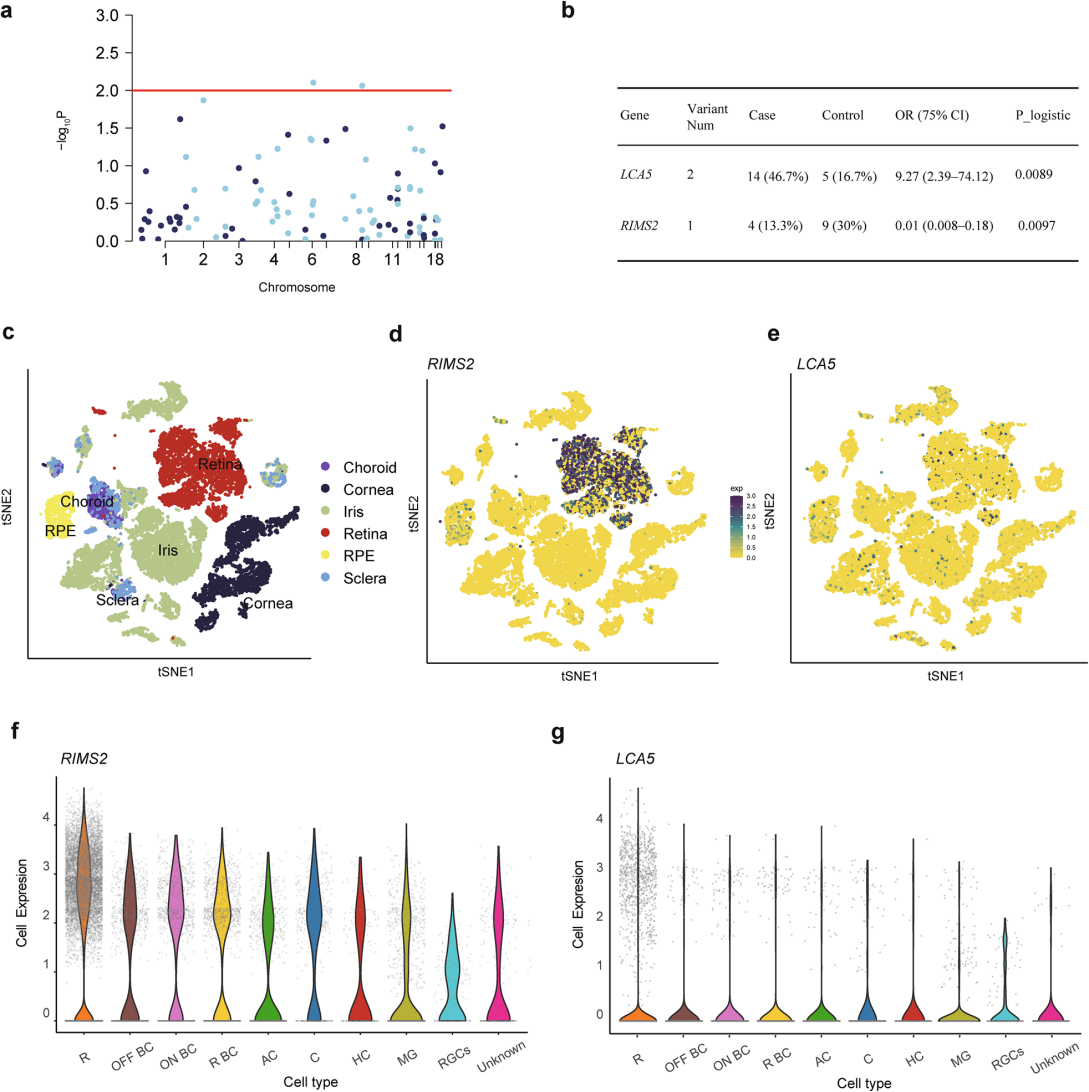
Collapsing analysis identifies two genes affecting the efficacy of orthokeratology. **a** Manhattan plots of the gene-based collapsing analysis. An excess of nonsynonymous genes within RetNet genes was tested using logistic regression; red line, P = 0.01. **b** Two genes from the collapsing analyses under the same model are shown, including the exact numbers of all qualifying cases and controls and the statistical calculations of association (OR and P). tSNE of (**c**) all tissue single-cell data with cells colored based on the expression of the (**d**) RIMS2 and (**e**) LCA5 genes. Gene expression levels are indicated by shades of blue. Violin plot of the expression of cell types in the retina for (**f**) RIMS2 and (**g**) LCA5. R, rod cells; OFF BC, OFF bipolar cells; ON BC, ON bipolar cells; R BC, rod bipolar cells; AC, amacrine cells; C, cone; HC, horizontal cells; MG, muller glia

To identify genes associated with the effect of orthokeratology lenses on myopia, an association analysis was performed in which individuals wer2e categorized based on the presence or absence of common nonsynonymous variants. The genes significantly associated with positive effects included three variants in *LCA5* (OR = 9.27, *P* = 0.0089) and *RIMS2* (OR = 0.01, *P* = 0.0097; Fig. 5a, b; Table 1). Cell-type specificity analysis of data from whole eyes consistently revealed that *RIMS2* was mainly expressed in the retina and that *LCA5* was expressed at low levels in various eye tissues (Fig. 5c, d, e). Furthermore, when the tissue was restricted to the retina, both genes presented the strongest expression in rod cells (Fig. 5f, g).

**Table 1:** All qualified variants in RIMS2 and LCA5 in 30 cases and 30 controls with ChinaMap MAF > 5%

Chromosome	Position	SNP	HGVS.c	HGVS.p	Variant type	PolyPhen	MAF in ChinaMap	Alleles in 30 cases	Alleles in 30 controls
*LCA5*									
chr6	79487131	rs1875845	c.1967G > A	p.Gly656Asp	Missense variant	Benign (0)	0.25	15	6
chr6	7951881	rs34068461	c.77A > C	p.Asp26Ala	Missense variant	possibly_damaging (0.808)	0.22	14	5
*RIMS2*									
chr8	104093618	rs55788818	c.3841C > T	p.Arg1281Cys	Missense variant	probably_damaging (0.998)	0.15	4	9

*MAF* = minor allele frequency; *SNP* = single nucleotide polymorphism

### Single variant association analyses in RetNet genes

All common variants of RetNet genes that passed standard quality control for association tests were then examined utilizing a generalized mixed-based method (SAIGE) capable of accommodating population structure, a sparse genetic relationship matrix and relatedness. The discovery analysis identified several variants that reached the significance level, including 16 SNPs (Table 2).

**Table 2:** The most significant single-variant associations on the effect of orthokeratology lenses identified by SAIGE analysis

SNP	A1	A2	Beta	SE	OR	*P* value	gene	Variant type
rs36006402	T	C	1.69	0.47	4.82	0.0003	*SLC7A14*	intron_variant
rs4785329	G	A	1.41	0.43	3.52	0.0012	*ZNF423*	intron_variant
rs2059479	A	C	2.02	0.65	6.20	0.0020	*TRPM1*	intron_variant
rs2285814	G	T	− 1.28	0.42	0.36	0.0021	*CLUAP1*	intron_variant
rs4655445	G	T	1.41	0.47	4.00	0.0028	*USH2A*	intron_variant
rs1227067	T	C	− 1.57	0.53	0.22	0.0031	*CDH23*	intron_variant
rs7097667	G	A	1.27	0.45	3.29	0.0050	*PDE6C*	intron_variant
rs7144028	A	C	− 1.33	0.49	0.32	0.0067	*TTC8*	downstream_gene_variant
rs28713337	G	A	1.44	0.53	3.97	0.0067	*HMX1*	downstream_gene_variant
rs9595937	A	G	− 1.15	0.43	0.36	0.0074	*RB1*	downstream_gene_variant
rs2812773	A	G	− 1.04	0.39	0.39	0.0075	*EYS*	intron_variant
rs3138137	C	A	− 1.42	0.53	0.29	0.0077	*RDH5*	intron_variant
rs12898728	T	C	1.38	0.52	3.30	0.0081	*NR2E3*	downstream_gene_variant
rs1886698	G	A	− 1.20	0.46	0.37	0.0087	*CDH3*	intron_variant
rs12661004	A	G	− 1.21	0.46	0.35	0.0091	*PRDM13*	upstream_gene_variant
rs2272854	C	G	1.19	0.46	2.85	0.0096	*TUBGCP6*	intron_variant

*SNP* = single nucleotide polymorphism; *SE* = spherical equivalent; *OR* = odds ratio

**Fig. 6:**
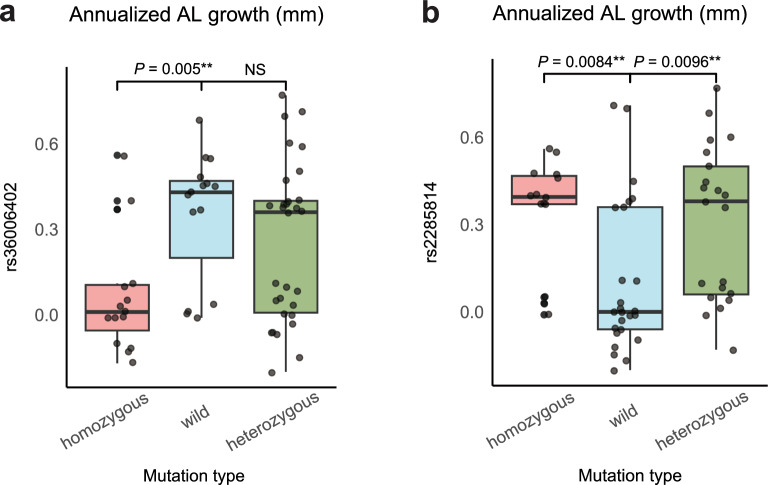
Cumulative difference in axial length (AL) growth. Differences in annual AL growth between groups carrying heterozygous, homozygous and wild-type variants of (**a)** rs36006402 and (**b)** rs2285814

The relationships between these 16 variants and the AL growth were then explored. The results revealed that, compared to those with the wild type, homozygous carriers of rs36006402 had lower AL growth (*P* = 0.005). Greater AL growth was found in both homozygous (*P* = 0.0084) and heterozygous (*P* = 0.0096) carriers of rs2285814 (Fig. 6).

## Discussion

In this study, one of the largest orthokeratology lens cohorts genotyped via WGS was compiled. Using this comprehensive dataset, we explored not only the clinical factors that influenced the effectiveness of orthokeratology lenses in controlling myopia through the annual increase in the AL but also the genetic landscape and underlying biological mechanisms of the efficiency of orthokeratology lenses.

Through rigorous and comprehensive data screening, 545 samples were retained out of a total of 1583 samples. The baseline data revealed a statistically significant negative correlation between age, initial myopia progression and the amount of ocular AL growth. Younger individuals with a lower baseline AL and SE experienced greater AL growth.

Age has been shown to be negatively correlated with AL growth after wearing orthokeratology lenses in previous studies [8, 17, 42–45]. This may be because eye axial elongation naturally decreases with age among children [46–49]. Moreover, younger myopic patients harbor more variants in genes that affect vision [50–52]. Several studies have also reported an association between higher baseline SE and lower amounts of ocular axial growth [15, 18, 53, 54]. Similarly, multiple studies have concluded that patients with higher baseline AL can retard axial growth while wearing orthokeratology lenses [55, 56]. A natural slowing of AL growth may occur once the eye approaches a specific threshold of myopia and AL. These findings also suggest that the use of orthokeratology lenses in older children with greater degrees of myopia may be more effective in slowing the progression of AL growth. On the other hand, for younger children with lower degrees of myopia, a combination of treatment methods, such as low-concentration 0.01% atropine[57–59], may be needed for optimal results, as previous research has shown that combined use is effective in younger children [45].

Through quartile division, a total of 143 patients whose annual AL growth was less than 0.09 mm and 140 patients with annual AL growth more than 0.33 mm were identified. These groups reflect a large difference in the degree of acceptance of orthokeratology lenses by the individuals in each group. A further 30 cases were selected from each group for WGS. There was a statistically significant difference in the amount of AL growth between both groups. A difference in the baseline AL was also found between them. Additionally, baseline AL was considered a factor that promoted the efficacy of orthokeratology lenses, according to the logistic regression analysis. This finding further validates the findings from the correlation analysis, suggesting that a more severe initial state of myopia might be a potential factor for achieving better outcomes with the subsequent use of orthokeratology lenses. Together, these findings indicate that the baseline AL can be used as a factor for assessing and predicting the effectiveness of myopia control in patients.

Subsequently, the genetic characteristics of the samples and their influences on the effectiveness of orthokeratology lenses were explored at the genetic level by WGS. Gene-set burden analysis of the RetNet gene set revealed that common nonsynonymous variants promoted the effectiveness of orthokeratology. Visual signals from the peripheral retina have a strong influence on eye growth [60, 61], and biological processes mediated by genetic variants in the RetNet gene set may affect the defocusing effect of orthokeratology lenses [62], thereby enhancing or preventing the inhibitory effect on AL growth produced by wearing orthokeratology lenses. In addition, mutations in genes in RetNet have been implicated in early-onset high myopia [51, 52, 63]. The myopic pathway caused by variants in the RetNet geneset may also lead to more pathologically early-onset myopia than late-onset [62] myopia, such that younger patients have even higher annual AL growth.

Subsequently, two genes were found to be associated with orthokeratology lens control. *RIMS2* was found to play a negative role in the control effect of orthokeratology lenses. *RIMS2* exhibited the highest expression in the retina among all ocular tissues. The maximum expression was detected in rod cells in the retina. In contrast, nonsynonymous variants of *LC5A* facilitated the effect of orthokeratology lenses. The expression of *LCA5* was also the highest in rod cells. *RIMS2* is the primary large *RIM* isoform found at photoreceptor ribbon synapses and is crucial for maintaining normal synaptic connections. Mutations in *RIMS2* may result in post-photoreceptor defects affecting both the cone and rod signaling pathways [64], foveal changes and inner retinal thinning [65]. The findings of this study suggest that *RIMS2* may influence the effectiveness of orthokeratology lenses by affecting retinal contrast changes, which play a crucial role in the retina’s ability to sense defocus [66]. This could be mediated through its involvement in synaptic neurotransmitter transmission in rod cells, which are sensitive to changes in retinal contrast [67]. These contrast changes could help signal the retina to adjust eye growth in response to the optical changes induced by orthokeratology lenses. The *LCA5* gene is associated with Leber congenital amaurosis (LCA), a hereditary retinal disease that severely affects vision. Mutations in the *LCA5* gene can lead to functional impairment and structural abnormalities in the retinal photoreceptor cells [68–70]. However, no phenotypic differences were detected between samples harboring these two gene variants and those without. There were no significant differences in age, SE and baseline AL. This might be due to the limited sample size. In the future, larger-scale data is needed to explore the relationships between phenotypes and molecular characteristics at the genetic level, in order to uncover the underlying control mechanisms.

At the SNP level, association analysis uncovered 16 mutations located in 16 different genes related to the effectiveness of orthokeratology lenses. These signals suggest that the effectiveness of orthokeratology lenses is linked to certain genetic characteristics. Among these 16 variants, rs36006402 and rs2285814 were found to be significantly associated with AL growth. Individuals carrying the homozygous rs36006402 variant showed decreased AL growth compared to those with the wild-type or heterozygous variant, whereas those carrying rs2285814 in both homozygous and heterozygous manners had increased AL growth. rs36006402 (OR = 4.8, *P* = 0.0003) is located in the intron area of the *SLC7A14* gene and is inherited in a recessive manner. *SLC7A14* plays an important role in retinal development and visual function [71]. rs2285814 (OR = 0.36*, P* = 0.0021) also occurs at an intron position in the *CLUAP*1 gene. *CLUAP1* is associated with the intraflagellar transport (IFT) complex B group of proteins and undergoes IFT in both invertebrates and vertebrates, which is associated with photoreceptor maintenance [72, 73]. Ultimately, further analysis is required to replicate and functionally validate these associations.

The main limitation of this work is the small sample size for candidate gene association analysis, which may have resulted in insufficient statistical power and biased effect size estimation. Additionally, the follow-up period in this study was limited to one year, which may not have captured long-term changes in AL or the sustained effects of orthokeratology lenses. Future studies with longer follow-up periods will provide valuable insights into the durability and long-term efficacy of orthokeratology. Another limitation is the study’s retrospective nature. As a result, during the recall process for additional genome-wide data collection, some samples were lost to follow-up. To address this, further prospective studies with comprehensive data collection and longer follow-up periods are needed to validate and expand upon these results.

Despite these limitations, this study has several noteworthy strengths. First, to date, no research has explored genetic associations with the effectiveness of orthokeratology, with studies only addressing differences in clinical data. This study employed a GWS strategy in the Chinese population, providing high-density coverage of noncoding regions and offering the opportunity to identify novel susceptibility loci. This first genetic study of orthokeratology lens effectiveness highlights a significant milestone in the field, offering a wealth of insights into the genetic underpinnings and clinical manifestations of the use and effectiveness of orthokeratology lenses for myopia control.

## Conclusion

Our findings indicated that age, baseline AL and baseline SE are clinical factors that affect the effectiveness of orthokeratology. Further, a WGS-based association study restricted to a retinal disorder gene set was conducted for the first time in a Chinese cohort. These findings not only enhanced the efficiency of array-based genetic studies for identifying both common and low-frequency susceptibility variants but also highlighted the genetic etiology of orthokeratology lens effectiveness. The results of this study will contribute to the refining of current heuristics for clinical decision-making for this complex treatment method.

## Supplementary Information

Supplementary material 1. Detailed methods of DNA purification and variant annotation.

Supplementary material 2: Figure S1. Standard quality control of 60 WGS samples. Boxplot of (a) sample mean depth, (b) sample mean genotype quality (GQ), (c) sample mean call rate for 60 samples. d Principal component analysis plot comparing 60 individuals with East Asian populations from the 1000 Genomes Project.

Supplementary material 3. Table S1. Participant demographics of 545 and 60 samples.

Supplementary material 4: Figure S2. Burden test on different types of variants. Burden analysis used logistic regression for (a) all type of variants (b) common variants and (c) rare variants between cases and controls. OR, odds ratio; CI, confidence interval.

## Data Availability

The data that support the findings of this study are available from the corresponding author upon reasonable request.

## Abbreviations

WGS: Whole-genome sequencing
SE: Spherical equivalent
OR: Odds ratio
AL: Axial length
SNPs: Single nucleotide polymorphisms
AF: Allele frequency
MAF: Minor allele frequency
VEP: Variant effect predictor
BWA: Burrows-Wheeler Aligner
CADD: Combined Annotation Dependent Depletion
SKAT: SNP-Set (Sequence) Kernel Association Test
GLM: Generalized linear model
scRNA-seq: Single-cell RNA-seq
CHB: China Beijing
CHS: China South
CDX: Chinese Dai in Xishuanagbanna
JPT: Japan
KHV: Korean
1KG: 1000 Genome Project
GVCF: Genomic variant call format
D: Diopters
OD: Oculus dexter
OS: Oculus sinister
Dk: Oxygen permeability
