# Supplementary material for: Associations between RetNet gene polymorphisms and the efficacy of orthokeratology for myopia control: a retrospective clinical study
Source: Eye Vis (Lond). 2025 Mar 17;12:13. doi: 10.1186/s40662-025-00426-4 (PMC11912624; doi:10.1186/s40662-025-00426-4)
Supplement: Supplementary file 1 — Supplementary material 1. Detailed methods of DNA purification and variant annotation. [file 40662_2025_426_MOESM1_ESM.docx]

**Additional File 1. Detailed methods of DNA purification and variant annotation.**

DNA purification for sequencing

Proteinase K solution (20 µL) was added to a 1.5 mL nuclease-free centrifuge tube, followed by the transfer of 200 µL of liquid sample and 200 µL of lysis/binding buffer. The mixture was vortexed briefly and incubated at 56°C for 10 minutes, with inversion every 3 minutes. Next, 200 µL of isopropanol and 20 µL of magnetic beads were added, and the mixture was incubated at 4°C on a rotating mixer for 10 minutes. After a brief centrifugation, the tube was placed on a magnetic rack for 1 minute to allow bead capture, and the supernatant was discarded. The beads were washed by adding 500 µL of wash buffer I, followed by mixing for 1 minute and brief centrifugation. The tube was again placed on the magnetic rack to remove the supernatant. This washing step was repeated twice with wash buffer II. Following the final wash, the tube was centrifuged briefly, and the supernatant was removed. The beads were air-dried for 3 minutes at room temperature, ensuring complete evaporation of ethanol. Finally, 100 µL of elution buffer was added, and the mixture was incubated at 56°C for 10 minutes with inversion every 3–5 minutes and completed the DNA purification and extraction.

Variant annotation and classification

In detail, nonsynonymous variants were classified as PTV variants and missensen varinat including following annotation terms: (1) PTV: "frameshift_variant", "splice_acceptor_variant", "splice_donor_variant", "stop_gained", "start_lost", "stop_lost", protein_altering_variantor SpliceAI > 0.5 & LoF=HC. (2) Missense: inframe_insertion, "inframe_deletion", "missense_variant". The synonymous variant was predicted as "synonymous_variant". Finally, non-coding was classified as "intron_variant", "intergenic_variant", "downstream_gene_variant", "3_prime_UTR_variant", "5_prime_UTR_variant", "mature_miRNA_variant", "regulatory_region_variant", "non_coding_transcript_exon_variant" and "upstream_gene_variant".
