# Supplementary figures and images for: Associations between RetNet gene polymorphisms and the efficacy of orthokeratology for myopia control: a retrospective clinical study
Source: Eye Vis (Lond). 2025 Mar 17;12:13. doi: 10.1186/s40662-025-00426-4 (PMC11912624; doi:10.1186/s40662-025-00426-4)

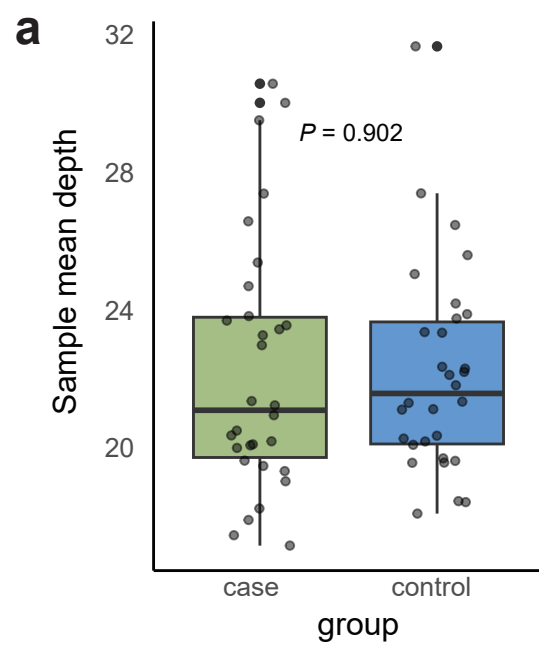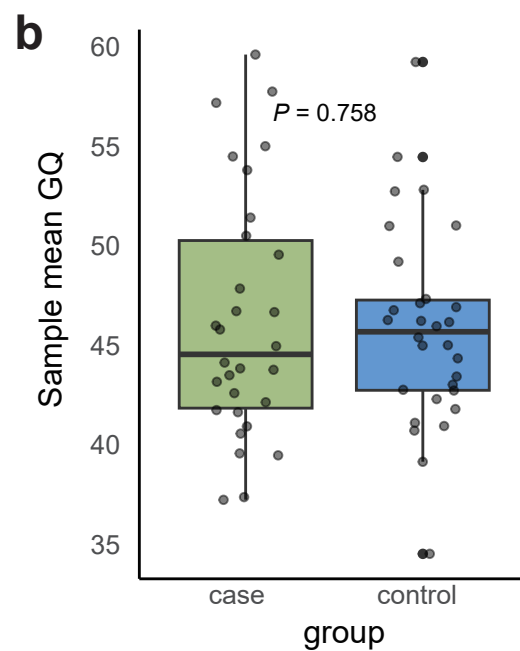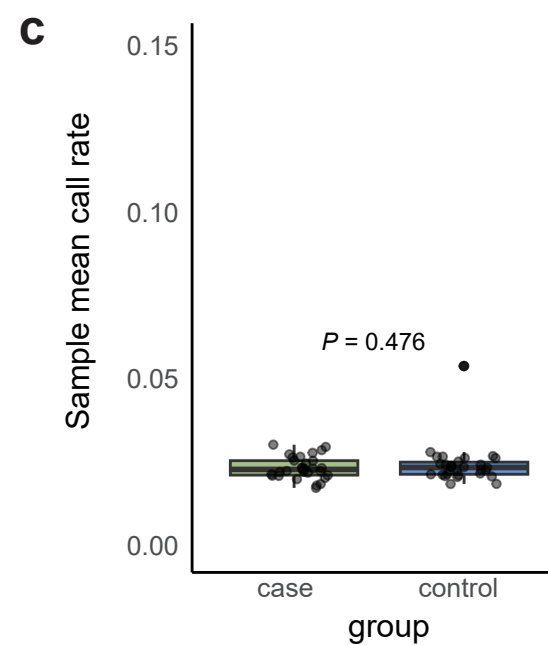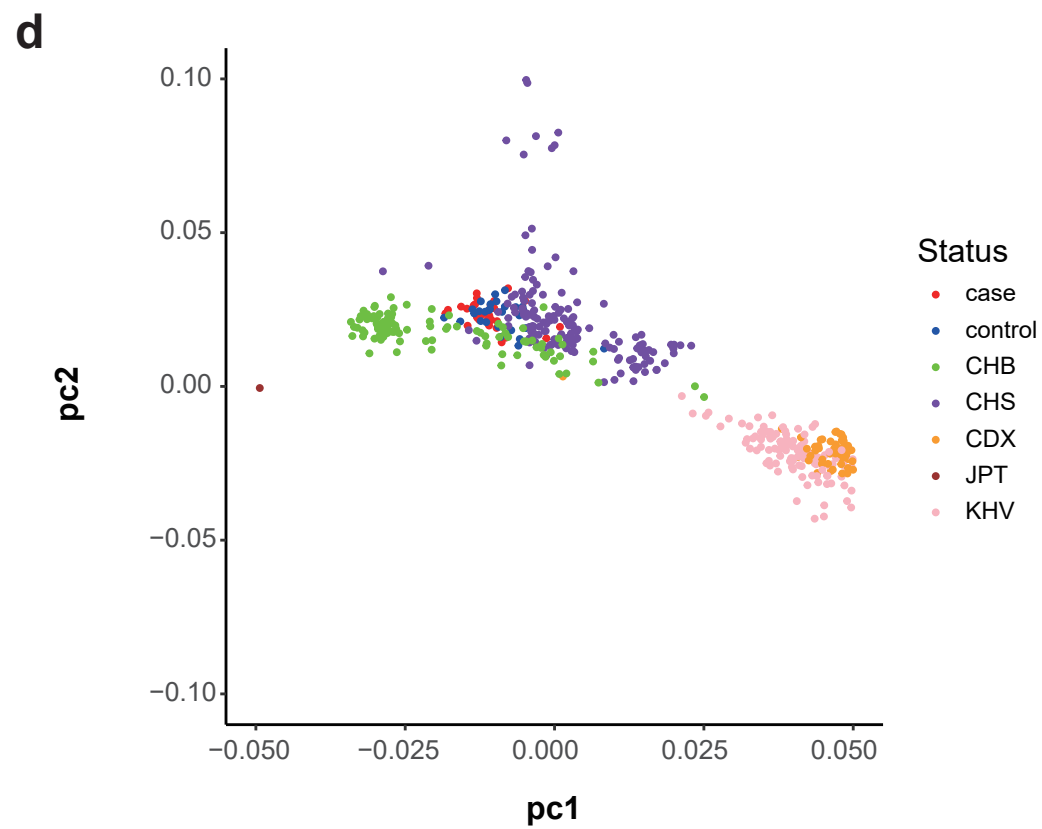

Supplement: Supplementary file 2 — Supplementary material 2: Figure S1. Standard quality control of 60 WGS samples. Boxplot of (a) sample mean depth, (b) sample mean genotype quality (GQ), (c) sample mean call rate for 60 samples. d Principal component analysis plot comparing 60 individuals with East Asian populations from the 1000 Genomes Project. [file 40662_2025_426_MOESM2_ESM.pdf]

**a**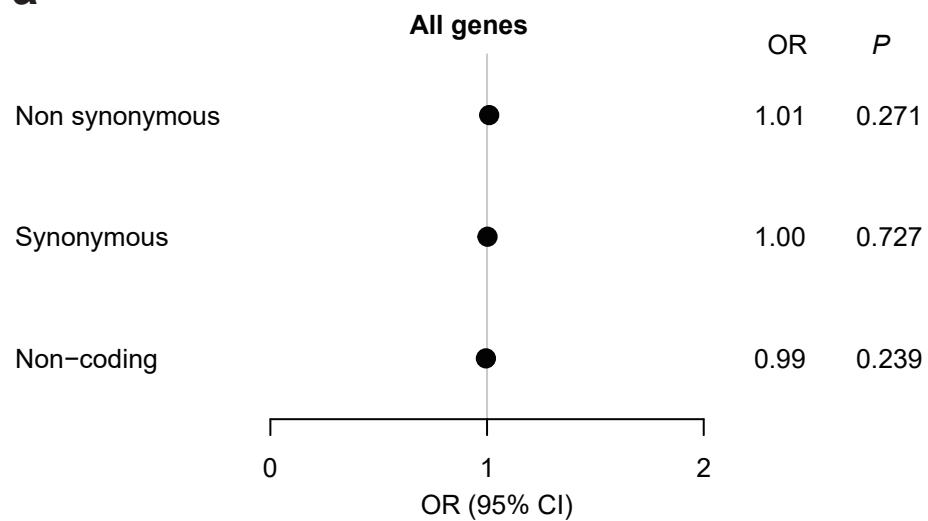**b**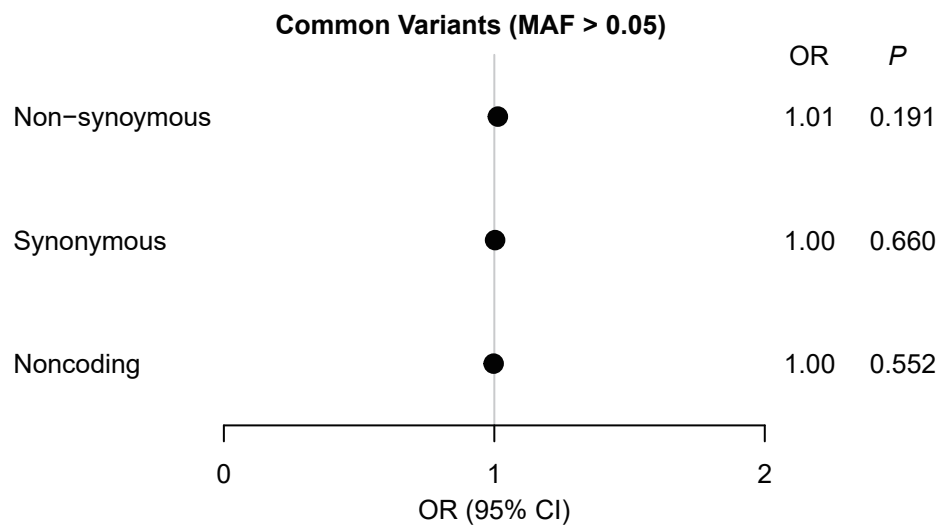**c**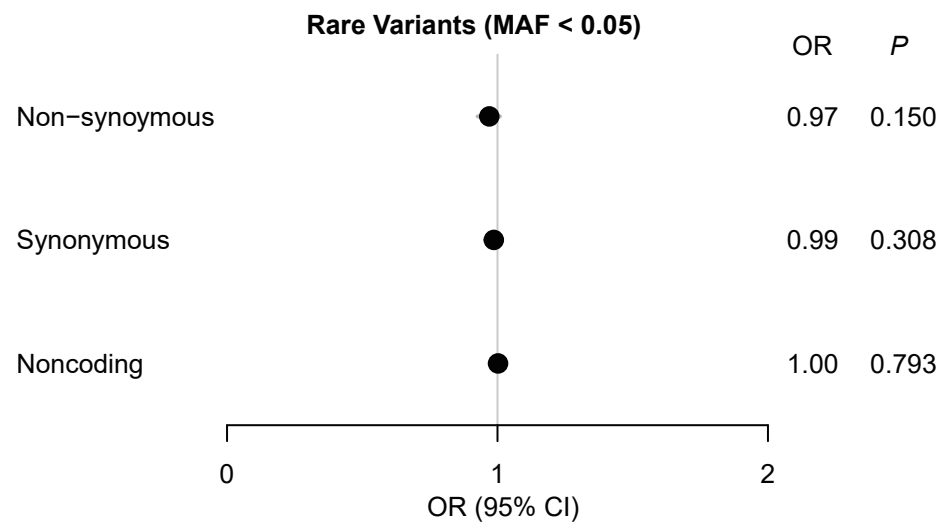

Supplement: Supplementary file 4 — Supplementary material 4: Figure S2. Burden test on different types of variants. Burden analysis used logistic regression for (a) all type of variants (b) common variants and (c) rare variants between cases and controls. OR, odds ratio; CI, confidence interval. [file 40662_2025_426_MOESM4_ESM.pdf]
