# Supplementary material for: Associations between RetNet gene polymorphisms and the efficacy of orthokeratology for myopia control: a retrospective clinical study
Source: Eye Vis (Lond). 2025 Mar 17;12:13. doi: 10.1186/s40662-025-00426-4 (PMC11912624; doi:10.1186/s40662-025-00426-4)
Supplement: Supplementary file 3 — Supplementary material 3. Table S1. Participant demographics of 545 and 60 samples. [file 40662_2025_426_MOESM3_ESM.docx]

**Additional File 3 Table S1.** Participant demographics of 545 and 60 samples.

| **Baseline demographic data** | **All**  **(n=545)** | **Completed (n=60)** | |
| --- | --- | --- | --- |
|  |  | **Case** | **Control** |
| Age at enrolment (years, mean ± SD) | 10.12 ± 1.27 | 9.27 ± 0.94 | 8.83 ± 0.83 |
| Male, % (n) | 55.78 (304) | 53.33 (16) | 33.33 (10) |
| Female, % (n) | 44.22 (241) | 46.67 (14) | 66.67 (20) |
| Axial length (mm, mean ± SD) | 24.85 ± 0.80 | 25.10 ± 0.67 | 24.51 ± 0.79 |
| Spherical equivalent (mean ± SD) | −3.11 ± 1.08 | −3.04 ± 1.00 | −2.63 ± 1.02 |
